# Dentition and Its Derangements

**Published:** 1863-04

**Authors:** A. Jacobi


### Selections.

DENTITION AND ITS DERANGEMENTS.—*By A. Jacobi, M. D. LECTURE XII.*—PART IV.—*Laryngismus Stridulus and Craniotabes.*

I have stated that the cause of laryngismus must be looked for in a nervous center; at all events there is no disease of any of the respiratory organs which exhibits similar symptoms, and post-mortem examinations have resulted in nothing that could explain these symptoms by any local alterations in the lungs or heart. Old Goelis already describes cases of mild laryngismus in connexion with chronic hydrocephalus. Keitel found, besides a hypetrophied and degenerated thymus gland, the skull soft, and its sutures and fontanels large, both the osseous cerebral tissues soft and hyperæmic, oblongated spine also soft, its membranes congested, and a tablespoonful of clear serum in the upper portion of vertebral canal. Marshal Hall once found the oblongated spine harder than normal; Evans made the observation of a child born with spina bifida, who would have an attack of laryngismus whenever the liquid of the sac was pressed into the vertebral canal. Caspari found the substance of the spine solid and white, and its dura mater much injected. The sinuses of the brain were filled with an enormous amount of black and thin blood, the substance of both the large hemispheres and cerebellum very soft. The phrenic nerve, moreover, was uncommonly hard, but the pneumogastric nerve "appeared more similar to the brain."

After all, the uniform presence of some alterations in the nervous centers appears to prove my first proposition, that laryngismus is the symptom of a deep-seated anomaly. In many cases congestion and inflammation of the membranes, especially the brain, have been found, together with their consequences, viz: more or less transudation. This process may take place very slowly indeed, and very generally does have a slow progress. Many cases of cerebral or meningeal effusion undoubtedly take place without laryngismus, but that laryngismus should occur without any affection of the nervous centres is more than merely doubtful. But there is one disease which appears to be the fundamental cause and origin of laryngismus. It is rhachitis. I merely refer you to a former lecture in order to remind you of these facts, that rhachitis is not only a disease of the osseous tissue, that originally, indeed it is the result of disorders in digestion and assimilation, and that impaired nutrition brings on anomalies in all the system thus intimately connected with rhachitis. Particularly it is the form of rhachitis which is found in nurslings which is apt to bring on severe and general symptoms, viz: the rhachitical softening of the cranial bones, or craniotabes, of which I have spoken in a former lecture. Craniotabes is always connected with the meningitic processes, effusion between the meninges, and into the brain and its ventricles; and thus its direct connexion with a large amount of cerebral effusion is easily understood.

Old authors, whose reports Elsaesser has collected in his book on "the soft occiput," although they did not understand the importance of the rhachitical softening of the parietal and occipital bones, relate a number of post-mortem examinations, and cases illustrating the subject. Of the cases of Kopp, one who died at ten months, had a very large fontanel, ununited sutures, and very flexible cranial bones; in another who died before the end of the fifth month, he mentions flexible cranial bones, and large fontanels. Caspari relates the case of a child, which was very large and fat, but always had "phlegm on his chest," and a large head, large fontanels, and swollen epiphyses; he adds, that the majority of his infants affected with laryngismus stridulus, had a rhachitical predisposition. Other writers accurately describe cases of craniotabes, the symptomatology of which I have given you in my lecture on the connexion of diseases of the bones with dentition. Thus Pegenstecher speaks of a child who was very large and fat, and was affected with convulsions in his seventh month, and afterwards with attacks of apnœa. Being sick so long, it grew emaciate and thin, and his skull had quite a peculiar, no longer spherical, but remarkably irregular and asymmetrical form. Hirsch found twice, a large head, and large fontanels. Keitel describes the attacks and body of a child who died in his twenty-second week, and had mostly ununited sutures; the small, triangular fontanel remained still open; the quadrangular was unproportionately large, and the skull soft and thin. Hachman has a similar report. In Gunther's child after weaning, "a true rhachitical constitution" developed itself, and gradually also the attacks of laryngismus. Landsberg also found the sutures open, and delays in protusion of the teeth In one case of Hauff's, all the cranial bones appeared of a dark blue color, and were so little ossified as to be easily cut by means of a knife and scissors, and so thin that the squamous parts of the temporal bones, and some parts of the parietal and occipital bones, had the thickness of good-sized paper. In another, the chest was very similar to the "chicken chest," and the commencement of rhachitis could not be denied. A child of Staub's had already in its first year the unmistakable symptoms of rhachitis, and had its first tooth at eighteen months.

Many such cases could be collected from literature; but those above taken from older authors, suffice to illustrate the connexion between craniotabes and laryngismus. It is true, however, that not every case of this affection must necessarily be the result of craniotabes. Elsaesser reports the case of a child who had his laryngismus brought on by hooping-cough, not before his craniotabes had healed; and there are a few cases of laryngismus in the second or third years, where craniotabes is generally no longer present. Thus other causes may bring it on; but do not forget, that nervous affections will oftentimes not disappear with the removal of their causes, and that together with craniotabes, alterations take place inside the cranium which are not so liable to heal as the affection of the osseous tissue itself; therefore, craniotabes may still be the cause of laryngismus, even where it appears to have entirely passed by. I hardly remember a case of my own, in which symptoms of general rhachitis and of rhachitical softening of the cranium were absent in laryngismus; thus this much is certain, that the majority of cases of laryngismus, or crowning inspiration, depend on craniotabes and general rhachitis. It is always the great predisposing cause, and thus the last and proximate causes of an attack of our disease, as we find them enumerated in the text books. such as fright, anger, cough, protrusion of a tooth, etc., are thus assigned their right place of but occasional and temporary importance. By the defective condition of the cranium the brain is more subject to external injuries, concussion, quick movements of the head, improper carrying on the arm, lying on a hard pillow, rocking, and high temperature both artificial and solar; and finally, we must not overlook the importance of such alterations as invariably take place, in rhachitis and craniotabes, in the nutrition of the system and the condition of the brain. At all events, you will hardly ever be mistaken in your etiology, when on examining a new case of laryngismus, you look for crantotabes. Whenever a child with laryngismus is brought to me, my first attention is given to the occiput and epiphyses, as my first prescription is almost invariably the regulation of diet and the use of iron.

